# Grafting the Way to the Systemic Silencing Signal in Plants

**DOI:** 10.1371/journal.pbio.0020224

**Published:** 2004-08-17

**Authors:** Kriton Kalantidis

## Abstract

Grafting is a powerful but complex means to study the spread of RNA silencing

Grafting is an ancient technique used by farmers and gardeners to combine desired attributes of the rootstock with those of the donor plant shoot, or scion. Grafting essentially saved European wine making: when the insect Dactylosphera vitifoliae devastated European grapewine varieties over the course of the late 1800s and early 1900s, the varieties were saved by grafting them onto resistant rootstocks from the New World. Since then, these rootstocks have been used to maintain the susceptible Old World cultivars. But grafting is also an excellent tool for scientists studying systemic signals traveling between the rootstock and distal parts of the plants, and vice versa. For example, two important studies (Palauqui et al. 1997; Voinnet et al. 1998) used grafting to demonstrate the spreading of RNA silencing in plants. However, it was a subsequent paper (Crete et al. 2001) that followed up on certain inconsistencies in the grafting results that pointed to subtleties important for both experimental design and understanding systemic signaling in plants.

RNA silencing (termed posttranscriptional gene silencing in plants, quelling in fungi, and RNA interference in animals) refers to the phenomenon whereby specific gene transcript levels are reduced in the presence of a related RNA. From studies of RNA silencing in several systems, much is now known about the mechanisms involved (Matzke 2002; Mlotshwa et al. 2002), but the systemic spreading in plants is still a bit of a mystery. Posttranscriptional gene silencing spreads systemically throughout the individual plants in a very characteristic manner reminiscent of viral spread. This has led to the hypothesis of a systemic silencing signal that is produced in the tissues where silencing is initiated and is then transmitted to the distant parts of the plant where it can initiate silencing in a sequence-specific manner. The sequence specificity of the silencing strongly implies that the signal is a nucleic acid, most likely an RNA, but the identity of the signal remains unknown. Silencing spreads mainly in the direction from carbon source to carbon sink, that is, from tissues such as leaves that export the sugar products of photosynthesis, to tissues such as roots that import these products, and it can take up to several weeks until it is established in the whole plant (Palauqui et al. 1997; Palauqui and Vaucheret 1998; Voinnet et al. 1998; Sonoda and Nishiguchi 2000).

As expected, the discovery of this process triggered a quest for the “systemic inducer” of the process: a signal that travels through the plant and is able to initiate silencing in a remote location within the plant. Grafting was an obvious tool to use in the quest for this signal, as it allowed silencing source and sink tissues to be of different origin.

Palauqui et al. (1997) were the first to unambiguously demonstrate that silencing spreads from a silenced rootstock to a nonsilenced scion. They used as a stock a transgenic tobacco carrying an additional copy of the endogenous nitrate reductase gene, *Nia*. Some of the transgenic lines generated always showed higher levels of *Nia* transcripts than the wild type—as expected from the presence of an additional gene—and were termed class I lines. However, other transgenic lines had undergone silencing for both the endogenous and exogenous *Nia* genes and those were termed class II lines. Pallaqui et al. (1997) found that silenced class II rootstocks were able to silence class I scions. This was true even in a “sandwich graft,” where a wild-type (nontransgenic) segment was grafted between the silenced stock and the nonsilenced scion. Spreading of silencing was unidirectional from stock to scion. Though not explicitly stated, it was implied that it took more than 3 wk after grafting for systemic silencing to occur in the scion. The reported rate of transmission was 100%.

Related experiments by Voinnet et al. (1998) used scions that transgenically expressed green fluorescent protein grafted onto plants with established silencing of the same transgene. There, too, silencing spread through the graft to the nonsilenced scion, even when a wild-type section was grafted between transgenic rootstock and scion. Unlike the *Nia* transgene, which has an endogenous counterpart in the wildtype plant, green fluorescent protein has no homolog in nontransgenic lines. Therefore, silencing spreading in the wild-type “spacer” in the sandwich grafts could not be assisted by an endogenous sequence. Rather, the systemic signal must have traveled all the way to the scion and induced gene silencing there. The establishment of systemic silencing took 4 wk in the “direct” grafts and 6 wk in the sandwich grafts. However, silencing spread to the scion only in some of the grafts: in ten out of 16 direct grafts and five out of 11 sandwich grafts.

I found these papers were very important not only for what they proved—the existence of a systemic signal of silencing—but also because they gave an unequivocal answer to the scientific questions they posed, using relatively simple methodology. Although excited by these successful examples of the transmission of silencing, I kept coming back to two questions: (1) what prevents transmission in some of grafts, and (2) why does it take longer to transmit silencing to the scion than it takes systemic silencing to reach the most remote parts of an intact plant?

When we started working on the silencing signal ourselves, we repeated some of the above experiments but found somehow lower efficiencies in the initiation of silencing in the scions. We soon realized that our results were influenced by the developmental stage of our scions. A paper from Jeff Meins's laboratory in Switzerland shed light on some aspects of the grafting puzzle. Researchers there introduced additional chitinase genes using bolistics, in sense or antisense orientation under the control of a strong promoter (35S) into chitinase transformant lines of tobacco that never exhibited spontaneous gene silencing (Crete et al. 2001). In lines bombarded late in plant development, triggering of silencing was rarely observed. However, when the transgene was introduced earlier in development, a large portion of the lines showed a substantial decrease and eventually full suppression of the chitinase mRNA levels. Lines that showed silencing were used as rootstocks and nonsilencing lines were used as scions in three types of grafting experiments. In the first type of grafting experiment, called top grafting, a 5-cm scion cut into a wedge at the bottom was inserted into the vascular ring at the cut surface of a 50-cm-high rootstock (Figure 1A). In the second type of experiment, reciprocal transverse grafts of 50-cm-tall plants were exchanged between class I and class II plants (Figure 1B). Finally, the third type of experiment involved plug grafts, which were made by exchanging transverse plugs of stems cut with a 5-mm-diameter cork borer from an internode approximately in the middle of a 50-cm plant (Figure 1C).[Fig pbio-0020224-g001]

**Figure 1 pbio-0020224-g001:**
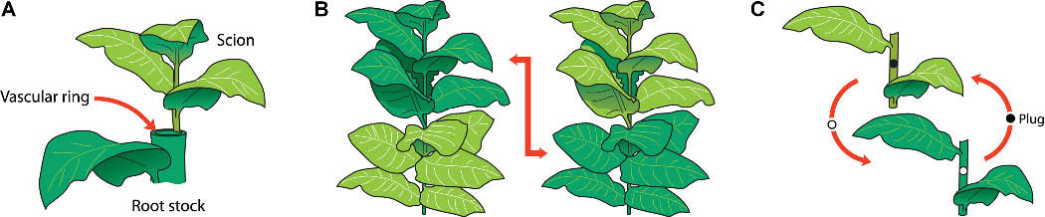
Grafting Method May Influence the Spreading Efficiency of the Silencing Signal (According to Crete et al. 2001) (A) Top grafting, the most effective in transmitting the silencing signal. (B) Reciprocal transverse graft. (C) Plug graft.

Surprisingly, only top grafting resulted in scions that were systemically silenced by a rootstock signal. Furthermore, even transmission after top grafting was less effective than expected; in one stock/scion combination only 27 out of 71 grafts exhibited transmission of the silencing signal. The authors also found that antisense-induced silencing was never transmitted to the scion. These findings do not answer the many questions about the mechanisms underlying systemic silencing, but they point us in certain directions.

The individual parts of a whole plant are, in terms of import and export, in an equilibrium that changes with development. When grafting takes place, how this equilibrium is altered depends on the individual “parts” that contribute to the “new” whole plant. In addition, there is now indirect evidence (Fagard and Vaucheret 2000) that what is source tissue and what is sink tissue in terms of sugar transport affects what is source and what is sink in terms of the systemic silencing signal. Taking into account the above findings and choosing the right combination of stock/scion, we have managed to significantly increase the efficiency of graft-transmitted silencing, a prerequisite for continuing the search for the systemic signal.

From the grafting experiments to date, it is now evident that the transporting capacity of the vascular tissue bypass that is formed at the graft junction does not fully reach the level of the original vascular tissue. The basis for these restrictions is not known. In a way, the graft interface functions as an unintentional filter. If the specificities of this “filter” were known, it would help us comprehend some transmission inconsistencies. Keeping in mind these limitations, grafting remains an invaluable tool in the search for the systemic silencing signal.

## Abbreviation

*Nia*: nitrate reductase gene
